# Inhibition of protein translation under matrix-deprivation stress in breast cancer cells

**DOI:** 10.3389/fmed.2023.1124514

**Published:** 2023-06-22

**Authors:** Shweta Warrier, Shivaani Srinivasan, Adithya Chedere, Annapoorni Rangarajan

**Affiliations:** Department of Developmental Biology and Genetics, Indian Institute of Science, Bengaluru, India

**Keywords:** protein translation, stress, matrix-deprivation, translatome, mTORC1, ISR, SUnSET, polysome

## Abstract

Matrix-deprivation stress leads to cell-death by anoikis, whereas overcoming anoikis is critical for cancer metastasis. Work from our lab and others has identified a crucial role for the cellular energy sensor AMPK in anoikis-resistance, highlighting a key role for metabolic reprogramming in stress survival. Protein synthesis is a major energy-consuming process that is tightly regulated under stress. Although an increase in protein synthesis in AMPK-depleted experimentally-transformed MEFs has been associated with anoikis, the status and regulation of protein translation in epithelial-origin cancer cells facing matrix-detachment remains largely unknown. Our study shows that protein translation is mechanistically abrogated at both initiation and elongation stages by the activation of the unfolded protein response (UPR) pathway and inactivation of elongation factor eEF2, respectively. Additionally, we show inhibition of the mTORC1 pathway known for regulation of canonical protein synthesis. We further functionally assay this inhibition using SUnSET assay, which demonstrates repression of global protein synthesis in MDA-MB-231 and MCF7 breast cancer cells when subjected to matrix-deprivation. In order to gauge the translational status of matrix-deprived cancer cells, we undertook polysome profiling. Our data revealed reduced but continuous mRNA translation under matrix-deprivation stress. An integrated analysis of transcriptomic and proteomic data further identifies novel targets that may aid cellular adaptations to matrix-deprivation stress and can be explored for therapeutic intervention.

## 1. Introduction

Breast cancer poses a severe challenge to global health as the leading cause of mortality in women (1). Breast cancer cells traverse the blood and lymphatic system and colonise distant organs like the lung, liver or bones and seed metastases, the major cause of cancer mortality (2). Metastatic cancer cells confront multiple stresses like hypoxia, nutrient deprivation, and oxidative stress. They additionally encounter matrix-deprivation, mechanical, and hemodynamic shear stresses in circulation (3). Therefore, stress-adaptation and survival are fundamental for cancer progression; deciphering these mechanisms holds promise for novel anti-cancer therapies.

Cell-matrix attachment provides crucial signals for growth, survival and maintenance of cellular homeostasis (4). Consequently, matrix-detachment triggers anoikis, a form of apoptosis (5). However, cancer cells attain anoikis-resistance via several mechanisms, including activating pro-survival signalling pathways and energy-conserving processes like autophagy and entosis (6–8). Recent studies have reported that dysregulation of metabolism during cellular acidosis, activation of autophagy and ROS generation in detached carcinoma cells promote anoikis-resistance (9–11). AMP-activated protein kinase (AMPK) is a metabolic energy sensor activated in response to multiple stresses (12). Pioneering work from our lab, as well as others, reported the activation of AMPK as a compensatory mechanism in detached cells to maintain energy homeostasis (8, 13–23).

Protein synthesis, which consumes nearly 30% of the total cellular energy (24), is one of the first processes to be inhibited as an adaptive stress-response (25). The shutdown of global mRNA translation under diverse stresses (glucose starvation, amino acid deprivation, hypoxia, and oxidative stress) happens through three major pathways involving the Integrated Stress Response (ISR)/Unfolded Protein Response (UPR) and mammalian Target of Rapamycin Complex 1 (mTORC1) pathway that inhibit initiation, and the AMPK-eEF2K axis that inhibits elongation (26). One study reported that in Ras-transformed mouse embryonic fibroblasts (MEFs), matrix-deprivation results in AMPK-mediated inhibition of the mTORC1 pathway, which conserves energy by suppressing protein synthesis (17). In yet another study, PERK-activation in matrix-detached cells was associated with autophagy and cell survival (27, 28). Additionally, another study on matrix-detached fibrosarcoma cells reported the activation of ISR, leading to cytoprotective autophagy (29). Yet, the cellular response of epithelial-origin cancer cells to matrix-detachment stress remains poorly understood.

In this study, we provide conclusive evidence that breast cancer cells respond to matrix-deprivation by activating multiple stress-response pathways and display a global reduction in protein synthesis. Additionally, we propose a putative alternate translatome that these cells could employ for resisting anoikis. These data begin to visualise matrix-deprivation as a legitimate stress-response and reprogramming of protein synthesis as an adaptive strategy for stress survival during metastasis.

## 2. Materials and methods

### 2.1. Cell lines and cell culture conditions

Breast cancer cell lines MDA-MB-231 and MCF7 (ATCC) were cultured in DMEM (Sigma-Aldrich) supplemented with 10% FBS (Invitrogen), penicillin (0.1 Ku/ml, Sigma), streptomycin (0.1 mg/mL, HiMedia) and incubated in a 5% CO_2_ incubator at 37°C. Primary murine mammary epithelial cells from littermate control and AMPKα1,α2 homo conditional Double Knockout (cDKO) mice were obtained as described previously (30) and used for SUnSET assay. To mimic matrix-deprivation (suspension cultures), cells were cultured on TCPS (35, 60, or 90 mm) dishes coated with a layer of 2% Noble agar to prevent deposition of ECM. Adherent cells were trypsinised, seeded onto the noble agar-coated dishes, and incubated for required periods.

### 2.2. Immunoblotting

Cells cultured in attachment or suspension conditions for 24 h were harvested using RIPA lysis buffer as described before (13). Adherent cells were lysed on petri dish while suspension cells were collected into microcentrifuge tubes, spun down to remove media and then lysed with lysis buffer. Equal amounts of proteins (30–50 μg), as measured by Bradford’s method, were resolved on SDS-PAGE gel and transferred to PVDF membrane. The membrane was blocked with 5% skimmed milk and probed overnight with primary antibodies (1:1000 dilution), followed by incubation with HRP-conjugated secondary antibodies (1:3000 dilution) and visualised with ECL substrate (Biorad). Densitometric analysis was done via ImageJ software.

Primary breast tissues were procured from Ramaiah Medical College (RMC) and Kidwai Memorial Institute of Oncology (KMIO), Bengaluru, India, in accordance with the institutional review board (IRB) of RMC and KMIO and in compliance with the Institutional Human Ethics Committee of IISc. Patient consent was acquired in writing before surgery. For tissue lysates, the ‘tumour tissues’ and ‘far away normal tissues’ (approximately 5 cm away from tumour boundary) were washed with 1X PBS. RIPA lysis buffer (80 μL for every 10 mg tissue) was added, and tissues were minced using homogeniser on ice within microcentrifuge tubes, and once separated from the debris, the lysates were quantified and taken for immunoblotting as described above. For patient-derived cell lysates, surgically resected breast tumour samples were enzymatically digested and cultured in monolayer condition. Once confluent, cells were trypsinised and further seeded in attachment and matrix-detached conditions for 24–48 h before being lysed for immunoblotting.

Several proteins were probed together in one run, but grouped separately for ease of understanding. Such blots share a common loading control (α-Tubulin) blot; this has been identified in figures.

### 2.3. Antibodies used in this study

Primary antibodies used in this study are p-mTOR^S2448^, p-Raptor^S792^, p-p70S6K^T389^, p-rpS6^S235/236^, p-4E-BP1^T37/46^, p-eIF2α^S51^, p-eEF2^T56^, mTOR, Raptor, p70S6K, rpS6, 4E-BP1, eIF2α, eEF2 (all from CST), CNBP (Abclonal), ATF4 (Abclonal), α-Tubulin (Calbiochem), β-actin (ThermoFisher Scientific), anti-Puromycin (DSHB). Secondary HRP-conjugated anti-rabbit and anti-mouse antibodies were obtained from Jackson ImmunoResearch Laboratories.

### 2.4. SUnSET assay

Cells cultured in attachment or suspension conditions for 24 h were treated with 1 μM puromycin (Puro, stock: 10 mM, Cayman Chemical) or vehicle control (H_2_O, ethanol) for 30 min. In the required control dishes, 100 μg/mL cycloheximide (CHX, stock: 100 mg/mL, Amresco) pre-treatment was given for 15 min, post which 1 μM puromycin was added for 30 min. Further, cells were lysed with RIPA lysis buffer and resolved in a gel. Immunoblotting protocol was followed as above with an added step of Ponceau S staining that served as the loading control.

### 2.5. Polysome profiling

Cells were cultured for 8 or 24 h in attachment or suspension cultures. Before harvesting, cells were pre-treated with 100 μg/mL CHX for 15 min. Lysates were prepared using Polysome Lysis buffer (PLB – 20 mM Tris-Cl, 200 mM KCl, 5 mM MgCl_2_, 1% NP-40, 1X protease inhibitor, 1 μL RNase Inhibitor and 100 μg/mL CHX). Equal RNA amounts (based on Nanodrop Lite) was layered onto 15–45% sucrose gradients and subjected to ultracentrifugation (40,000 rpm, 1:45 h, 4°C; Beckman LE-70) in SW-Ti 41 rotor (Beckman Coulter). Profiling was done in polysome fractionator (Biocomp Gradient Station ip), which provided the values for absorbance at 260 nm. The absorbance values were filtered at 0.6 or 1 Absorbance Unit to generate the required profiles using Graphpad Prism. For protocols involving puromycin, CHX was replaced with puromycin (100 μg/mL) in washing solution, PLB and also added to cells 10 min prior to treatment with CHX. For protocols involving EDTA, it was directly added into the lysate after centrifugation at the concentration of 100 mM.

### 2.6. Integrated analysis of transcriptomics and proteomics data on MDA-MB-231 cells

MDA-MB-231 cells harbouring pTRIPZ vector, where shRNA is induced only upon doxycycline treatment, were generated previously (31). These cells without doxycycline induction were used in the current study for integrated omics analyses. Their transcriptomes and proteomes were obtained by subjecting them to RNA Sequencing (Genotypic Technology Pvt. Ltd., Bengaluru) and LC-MS/MS (Institute of Bioinformatics (IOB), Bengaluru) analysis, respectively. Only genes with RNA-matched proteins were considered for this analysis. *Z*-score normalisation was performed separately on the Log_2_ (Fold change values) for transcripts and proteins. *Z*-score value of ±2 was taken as the threshold to determine differentially expressed transcripts and proteins. To obtain an enriched translatome (candidates that were differentially translated at protein level yet remained unchanged at transcript level), we considered the genes belonging to the proteome and transcriptome with differential protein expression (|*Z*-score| ≥ 2) and having non-differential transcript expression (|*Z*-score| < 2). Morpheus-Broad Institute software was used to represent the data as heatmaps.^1^

### 2.7. Statistical analysis

Statistical analysis of western blots was done by performing Student’s paired *t*-test using the Graphpad Prism v8.0.2 software. All data are represented as mean ± S.E.M of three biologically independent experiments unless stated otherwise. *p*-values <0.05 were considered significant. * represents value of *p* <0.05, ** represents value of *p* <0.01, *** represents value of *p* <0.001, and **** represents value of *p* <0.0001.

## 3. Results

### 3.1. Matrix-deprivation stress inhibits core components of the translation machinery

Translation, the cellular synthesis of proteins, is a high energy-demanding process that is stalled during stress to restore energy homeostasis and aid cell survival (24, 32). AMPK is a stress-sensor kinase that attenuates bioenergetic stress by activating multiple stress-response pathways (33). In our current study, in matrix-deprived MDA-MB-231 breast cancer cells, we observed the activation of AMPK via phosphorylation of Thr172 residue and also functionally by the phosphorylation of its *bona fide* substrate ACC (Acetyl-CoA carboxylase) at Ser79 residue (Supplementary Figure S1A). Cells mediate stress response via multiple pathways, among which Unfolded Protein Response (UPR) converging at eIF2α, and mTORC1 pathway are involved in the regulation of translation initiation, while the AMPK-eEF2K axis mediates regulation of translation elongation (26). To study which of these stress-response pathways are activated during matrix-deprivation, we cultured MDA-MB-231 and MCF7 breast cancer cells on Noble agar-coated tissue culture plates (henceforth referred to as ‘suspension’ condition) that prevent deposition of ECM proteins and thus prevent cell adhesion.

**Figure 1:**
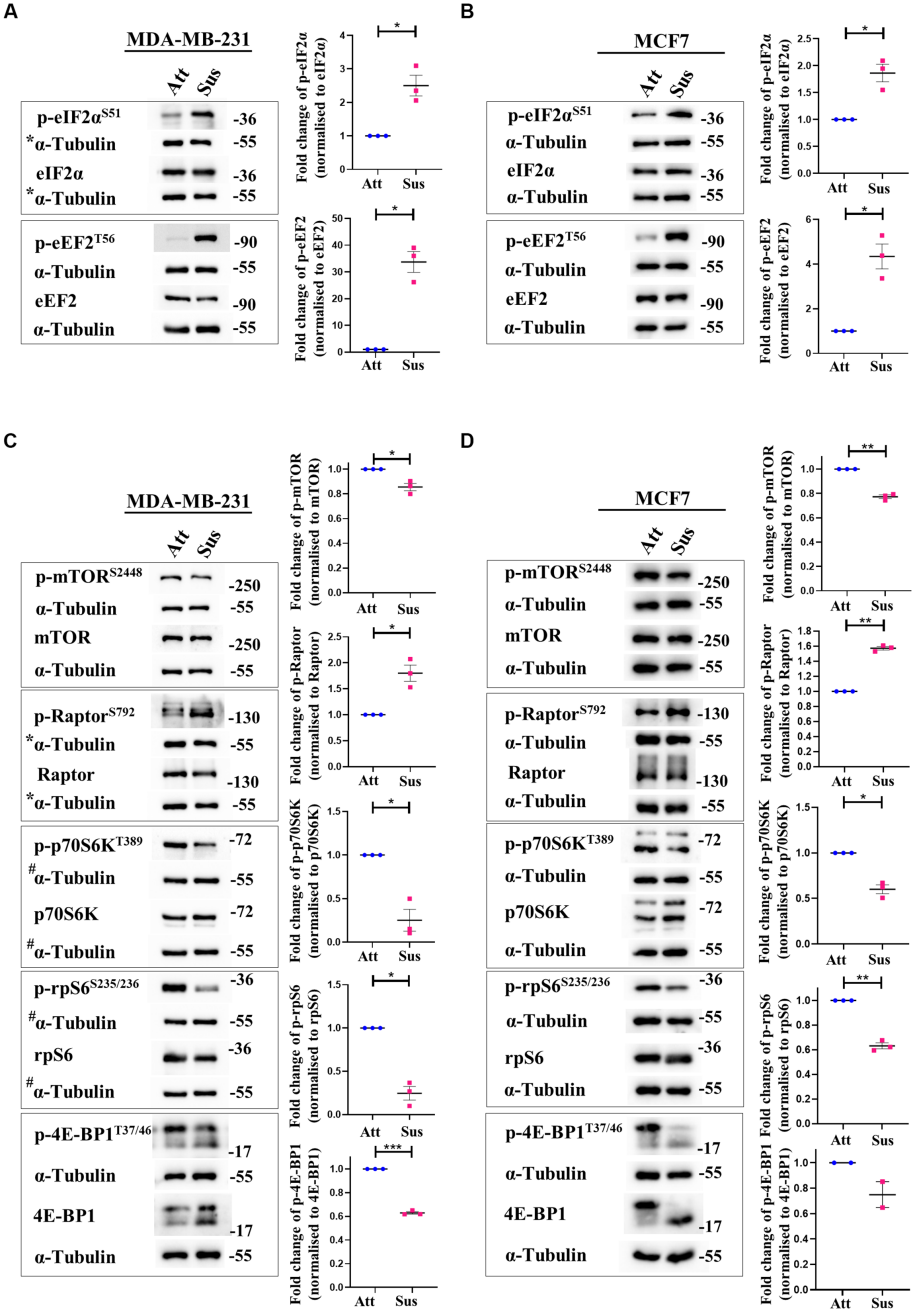
Matrix-deprivation stress regulates multiple translation factors to inhibit protein synthesis. Representative immunoblots of the following cell lysates were probed for the specified proteins. **(A)** and **(B)** MDA-MB-231 **(A)** and MCF7 **(B)** cells cultured in attachment (Att) and suspension (Sus) for 24 h were harvested for immunoblotting. Representative immunoblots show p-eIF2α and p-eEF2 levels along with their total protein levels. **(C)** and **(D)** MDA-MB-231 **(C)** and MCF7 **(D)** cells cultured in attachment (Att) and suspension (Sus) for 24 h were harvested for immunoblotting. Representative immunoblots show p-mTORC1, p-Raptor, p-p70S6K, p-rpS6, and p-4E-BP1 levels along with their total protein levels. Multi-panel blots for phospho (p) and total proteins were assembled by running the same lysates in duplicates in the same run, with α-tubulin as a loading control for each duplicate. After densitometric analysis of blots, relative phospho-protein levels were obtained by normalisation to individual loading control (α-Tubulin) and then respective total protein levels. Graphs represent densitometric quantification of the immunoblots. Error bars represent mean ± S.E.M; *n* = 3 (MCF7 immunoblot of 4E-BP1; *n* = 2). The asterisks indicate statistical significance as determined by Student’s paired *t*-test (* = *p* < 0.05, ** = *p* < 0.01, and *** = *p* < 0.001). *The blots for p-eIF2α/eIF2α in Figure 1A and p-Raptor/Raptor in Figure 1C share the same loading control (α-Tubulin) as they were probed together but grouped independently for ease of understanding. ^#^The blots for p-p70S6K/p70S6K and p-rpS6/rpS6 in Figure 1C share the same loading control (α-Tubulin) as they were probed together but grouped independently for ease of understanding.

In response to stresses like hypoxia and nutrient deprivation, distinct stress-responsive kinases catalyse the phosphorylation of eukaryotic initiation factor 2α (eIF2α) at residue Ser51 and inhibit translation initiation by impacting ribosome recruitment (34, 35). Additionally, translation elongation is also compromised through the inactivating phosphorylation of eukaryotic elongation factor 2 (eEF2) at Thr56 by the AMPK-eEF2K axis (36). Thus, we measured the phosphorylation of eIF2α and eEF2 at these specified residues in matrix-detached cells compared to their adherent counterparts. In suspension condition, we observed remarkable hyperphosphorylation of eIF2α and eEF2 in MDA-MB-231 (Figure 1A) and MCF7 (Figure 1B) cells. The total levels of these proteins remained unchanged in both conditions (Figures 1A,B). These data suggest that protein translation initiation and elongation are both inhibited in matrix-deprived breast cancer cells.

Signalling through the mTORC1 pathway positively regulates translation initiation and elongation by controlling various components of the translational machinery (37). Thus, we systematically examined the mTORC1 pathway in attached and matrix-detached breast cancer cells. Phosphorylation of mTOR at Ser2448 is required for its kinase activity (38). Under conditions of stress, there is a reduction in the Ser2448 phosphorylation due to AMPK-mediated activation of TSC2 (39, 40). Similarly, Raptor, a constituent of the mTORC1 complex, is directly phosphorylated at Ser792 by AMPK. This renders it incapable of binding to form the active complex, and thus the activity of mTORC1 is inhibited (41). Hence, we measured the phosphorylation of both these proteins in matrix-detached cells. In suspension conditions, we observed a significant decrease in the phosphorylated levels of mTOR and an increase in the phosphorylated levels of Raptor compared to attached cells (Figures 1C,D; 1st panel and 2nd panel, respectively) in both cell types.

Inactivation of mTORC1 leads to the inhibition of its downstream effectors—p70S6K and 4E-BP1. Inactive mTORC1 fails to phosphorylate its downstream target p70S6K at Thr389, leading to its inhibition (42). Inhibited p70S6K, in turn, fails to phosphorylate rpS6 at Ser235/236, compromising its involvement in ribosome biogenesis (43, 44). In line with our above observation of mTORC1 complex inhibition, we observed a reduction in the phosphorylated levels of p70S6K and rpS6 in matrix-detached cells (Figures 1C,D; 3rd and 4th panel). Another direct substrate of mTORC1 is 4E-BP1, which sequesters 5’ mRNA cap-binding eukaryotic initiation factor 4E (eIF4E) and inhibits translation initiation (45, 46). Inactivation of mTORC1 complex reduces the phosphorylation of 4E-BP1 (47). Consistent with this, we observed a reduction in the phosphorylated levels of 4E-BP1 in suspension conditions (Figures 1C,D; 5th panel). The total protein levels for all these proteins remained unchanged.

Thus, immunoblotting of multiple mTORC1 core proteins and its downstream targets (mTOR, Raptor, p70S6K, rpS6 and 4E-BP1) revealed that the mTORC1 pathway is inhibited in matrix-deprived breast cancer cells (Figures 1C,D) which is suggestive of inhibition of global protein translation under matrix-detached stress.

To understand the translational relevance of this result in clinical settings, analysis of stress-response pathways in circulating tumour cells (CTCs)—the proponent of metastasis—would be ideal. To mimic the same, we performed an *ex-vivo* approach to study the stress-response pathways activated by matrix-deprivation in human breast cancer patient-derived cells. Consistent with established cell lines, we observed the inhibition of translation initiation in matrix-deprived cells by the activation of ISR pathway (p-eIF2α^Ser51^) and the repression of the mTOR pathway (p-Raptor^Ser792^, p-rpS6^Ser235/236^). We also obtained similar results for inhibition of translation elongation via the AMPK-eEF2 pathway (p-eEF2^Thr56^) (Supplementary Figure S1B). Altogether, the results from patient-derived cells further substantiate our observations from breast cancer cell lines.

Taken together, our results suggest that protein synthesis is abrogated through multiple stress-response pathways in matrix-deprived breast cancer cells.

### 3.2. Residual translation retained in matrix detachment despite inhibition of global protein synthesis

**Figure 2:**
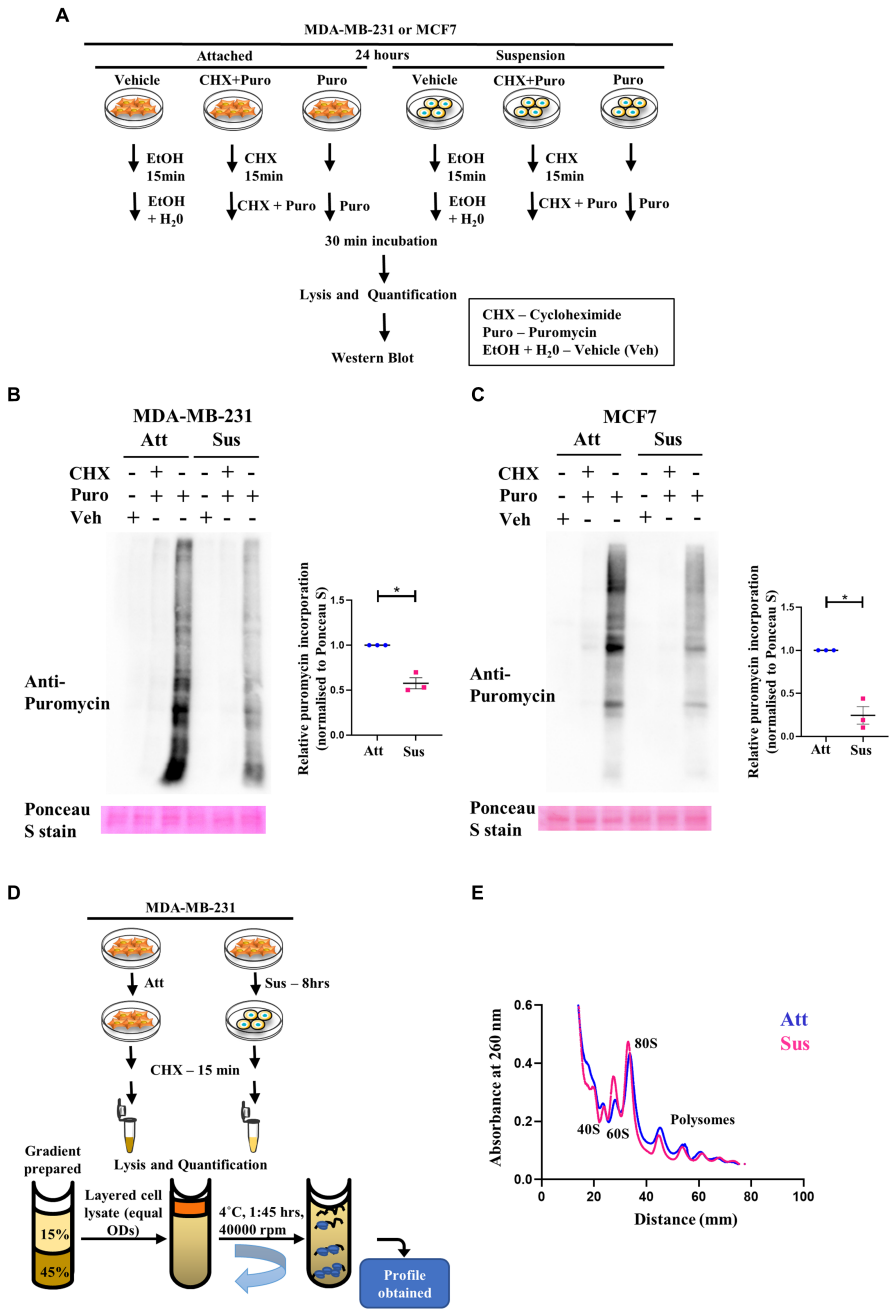
Residual translation retained in matrix detachment despite inhibition of global protein synthesis. **(A)** Schematic representing the basic steps used in the SunSET assay. **(B)** and **(C)** SunSET Assay to determine translational activity in MDA-MB-231 **(B)** and MCF7 **(C)** cells cultured in attachment (Att) and suspension (Sus) for 24 h. Cells were pre-treated with vehicle control (Veh), cycloheximide (CHX), and puromycin (Puro) for the specified time points, then harvested for immunoblotting. Representative immunoblot probed for anti-puromycin, quantified and normalised to respective Ponceau S levels. Graphs represent densitometric quantification of the immunoblots. Error bars represent mean ± S.E.M; *n* = 3. The asterisks indicate statistical significance as determined by Student’s *t*-test (* = *p* < 0.05). **(D)** Schematic representing the basic steps used in Polysome Profiling. **(E)** Polysome profiling for measuring translational distribution of ribosomes in MDA-MB-231 cells cultured in attachment (Att) and suspension (Sus) for 8 h. Cells were pre-treated with cycloheximide (CHX) for 15 min, then harvested for loading on 15–45% sucrose gradients. Following ultracentrifugation, polysome profiles for Att (Blue) and Sus (Pink) conditions are superimposed using the Graphpad Prism software to display combined profiles of attachment and suspension (*n* = 3). The peaks containing the small ribosomal subunit (40S), the large ribosomal subunit (60S), the monosome (80S), and polysome peaks are indicated on the profile.

Our results above revealed a concurrent inhibition of translation initiation (eIF2α) and elongation (eEF2) factors, as well as the mTORC1 pathway in response to matrix-deprivation. Inhibition of these pathways is expected to repress global protein synthesis (48, 49). Thus, to functionally characterise the extent of this inhibition, we employed the SUnSET (SUrface SEnsing of Translation) assay to assess the relative translational output of matrix-deprived MDA-MB-231 and MCF7 cells compared to their adherent counterparts (schematic in Figure 2A). Incorporation of puromycin into elongating peptides, followed by immunodetection, directly measures the rate of global protein synthesis (50). As expected, in adherent cells, pre-treatment with cycloheximide (translation inhibitor) completely abolished puromycin incorporation into nascent peptides (Figures 2B,C). Furthermore, cells treated with the vehicle control alone displayed no puromycin signal, confirming the specificity of the antibody. Interestingly, in cells subjected to matrix-detachment for 24 h, we observed a significant reduction in puromycin incorporation in MDA-MB-231 (Figure 2B) and MCF7 (Figure 2C) cells. Since suppression of global protein synthesis is a critical readout of cellular stress response (48), our results corroborate that, as with other stresses (26, 51–53), the stress of matrix-deprivation also suppresses global protein translation. Further, although multiple components of the translational machinery of protein synthesis are inhibited (as indicated in Figure 1), we observed depleted puromycylation signals in the suspension lanes (Figures 2B,C, last lane), indicating that minimal translation was still sustained.

Additionally, it is known that activation of AMPK under cellular stresses leads to inhibition of anabolic processes like protein translation for conservation of energy (26). To check if the inactivation of AMPK perturbs protein translation, we performed SUnSET assay on AMPKα1,α2 homo cDKO and AMPKα1,α2 double-floxed littermate control murine mammary epithelial cells. We observed increased puromycylation of peptides in AMPK cDKO cells in 24 h (Supplementary Figure S2A). This shows that the regulation of global protein translation is abrogated in the absence of AMPK, highlighting the importance of AMPK in maintaining cellular energy homeostasis.

Another method to assay global protein translation is to functionally measure the bulk translational activity by polysome profiling (54). Rapid protein synthesis relies upon formation of polysomes—actively translating ribosomes bound to an mRNA (55). A schematic of the major steps in the protocol of polysome profiling is depicted in Figure 2D. Polysome profile of MDA-MB-231 attached cells showed well-segregated peaks for 40S, 60S, 80S ribosomes (monosomes) and polysome peaks (Supplementary Figure S2B, top panel). To confirm the integrity of these peaks, we also treated the attached MDA-MB-231 cells with EDTA and puromycin, two different types of translation inhibitors. As expected, we observed that the peaks of monosomes and polysomes disappear in the presence of EDTA, and the peaks of polysomes disappear upon puromycin treatment (Supplementary Figure S2B, middle and bottom panels). While capturing early translational changes upon 8 h of matrix-deprivation in MDA-MB-231 cells, we observed an increase in the 60S peak suggesting a disassembly of ribosomal subunits (Figure 2E). We also observed a reduction in polysome peaks upon matrix-deprivation with a concomitant shift of ribosomes into the monosomal fraction (80S peak) suggestive of an initiation block on translation (Figure 2E). Similar results were also seen in the polysome profile upon 24 h of matrix-deprivation with the difference in translational activity more pronounced by this time-point (Supplementary Figure S2C). This result corroborates our previous observation of the SUnSET assay, both of which indicate a reduction in global translation in suspension conditions. Interestingly, it is important to note that the polysome peaks did not completely disappear upon encountering matrix-detachment stress (Figure 2E; Supplementary Figure S2C). These results together suggest that non-canonical translation mechanisms might be operating as an adaptive stress-response strategy to ensure cell survival.

This prompted us to check the levels of Activating Transcription Factor 4 (ATF4), which is preferentially translated by the activation of ISR pathway (56). ATF4 promotes stress endurance in cancer cells by regulating the expression of multiple stress response genes (57). We observed an increased level of ATF4 under matrix-deprived conditions in both breast cancer cell lines (Supplementary Figure S2D).

Taken together, these results confirm that matrix-deprivation inhibits global protein translation by the induction of multiple stress-response pathways.

### 3.3. Identification of differential translatome in matrix-deprived breast cancer cells

**Figure 3:**
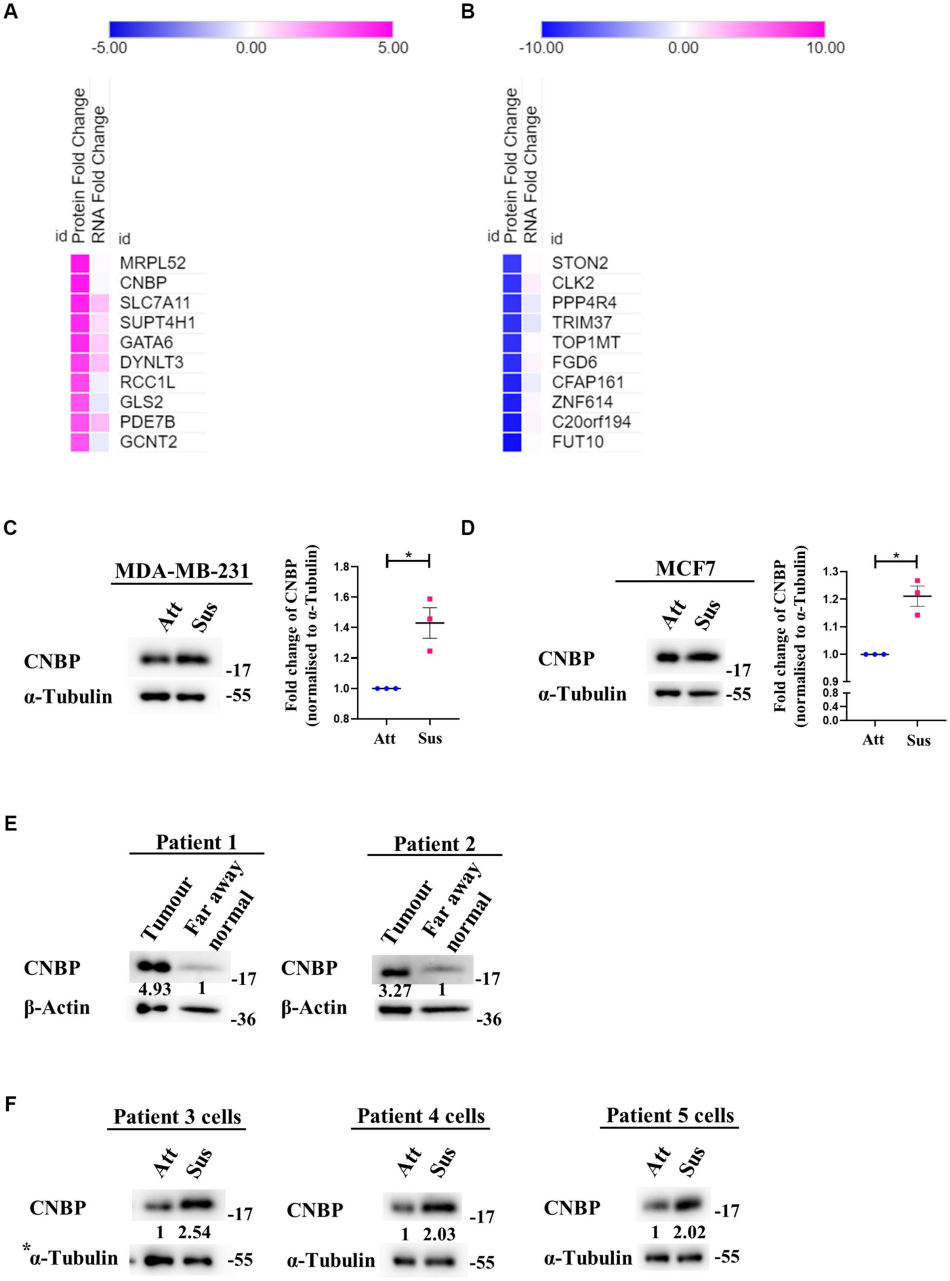
Identification of a differential translatome in matrix-deprived breast cancer cells. Comparison of transcriptomics and proteomics data of MDA-MB-231 cells, cultured under attached and suspension culture conditions for 24 h. **(A)** and **(B)** Heat map depicting protein and mRNA expression profiles from top 10 upregulated and downregulated proteins in MDA-MB-231 cells cultured in adherent and suspension condition for 24 h. Heat map also depicts their non-significant changes in mRNA expression levels. Upregulated proteins in **(A)** are shown in ‘pink’ and downregulated proteins **(B)** are shown in ‘blue’ for suspension condition as compared to the attached condition. **(C)** Representative immunoblot for CNBP expression levels in MDA-MB-231 cells cultured in attachment and suspension conditions for 24 h (*n* = 3). **(D)** Representative immunoblot for CNBP expression levels in MCF7 cells cultured in attachment and suspension conditions for 24 h (*n* = 3). After densitometric analysis of blots, relative protein levels were obtained by normalisation to individual loading control (α-Tubulin). Graphs represent densitometric quantification of the immunoblots. Error bars represent mean ± S.E.M. The asterisks indicate statistical significance as determined by Student’s *t*-test (* = *p* < 0.05, **** = *p* < 0.0001). **(E)** Representative immunoblots for CNBP expression levels in breast cancer patient far away normal and tumour tissues (*N* = 2). After densitometric analysis of blots, relative protein levels were obtained by normalisation to individual loading control (β-Actin) and then respective far away normal tissue protein levels. **(F)** Representative immunoblots for CNBP expression levels in patient-derived breast cancer cells cultured in attachment (Att) and suspension (Sus) for 24–48 h (*N* = 3). After densitometric analysis of blots, relative protein levels were obtained by normalisation to individual loading control (α-Tubulin). *The blots of CNBP in Figure 3F, 1^st^ panel and p-Raptor/p-rpS6 in Supplementary Figure S1B, 3^^rd^^ panel share the same loading control (α-Tubulin) as they were probed together but grouped independently for ease of understanding.

Despite suppression of protein synthesis, reprogramming of translational machinery to synthesise stress-adaptive translatomes is well-known (58). Usually, alternate translation profiles are generated when translational efficiency of mRNAs is prioritised over mRNA copy number variations (59). Our previous result (Figure 2) indicated residual translation despite global repression of protein synthesis under matrix-deprivation. This prompted us to investigate the altered translatome of matrix-detached breast cancer cells. For the same, we compared the transcriptomics and proteomics data of MDA-MB-231 cells in attached and suspension culture conditions for 24 h. Preliminary data analysis identified a few proteins that are upregulated or downregulated in suspension condition with no significant change in their mRNA levels, which are listed in Figures 3A,B, respectively. Some of these proteins and their biological functions are also detailed in Supplementary Tables S1, S2. Similar evidence on altered translatomes has also been listed in multiple other stress-related studies (51–53, 60).

One protein that showed upregulation at the protein level with no corresponding change in transcripts is CCHC-type Zinc finger Nucleic acid Binding Protein (CNBP), known to promote proliferation and chemoresistance in cancer (61–65). CNBP has both transcriptional and translational roles, notably acting as an ITAF (IRES Trans-Activating Factor) in medulloblastoma (62, 66). It has also been suggested to be involved in mRNA metabolism during stress (67). We observed a significant increase in the levels of CNBP in both matrix-deprived MDA-MB-231 (Figure 3C) and MCF7 (Figure 3D) cells. Additionally, immunoblotting of primary tumours from breast cancer patient samples showed increased levels of CNBP as compared to far away normal tissues (Figure 3E). Furthermore, increased levels of CNBP were also obtained in matrix-deprived patient-derived breast cancer cells (Figure 3F). Together, these results suggest that CNBP may play an essential role in stress adaptation and cancer progression.

Altogether, our results warrant matrix-deprivation as a stress experienced by cancer cells that leads to suppression of protein synthesis via several stress-response pathways. We also present evidence for the existence of an altered translatome aiding cancer cell survival in matrix-detached condition.

## 4. Discussion

**Figure 4:**
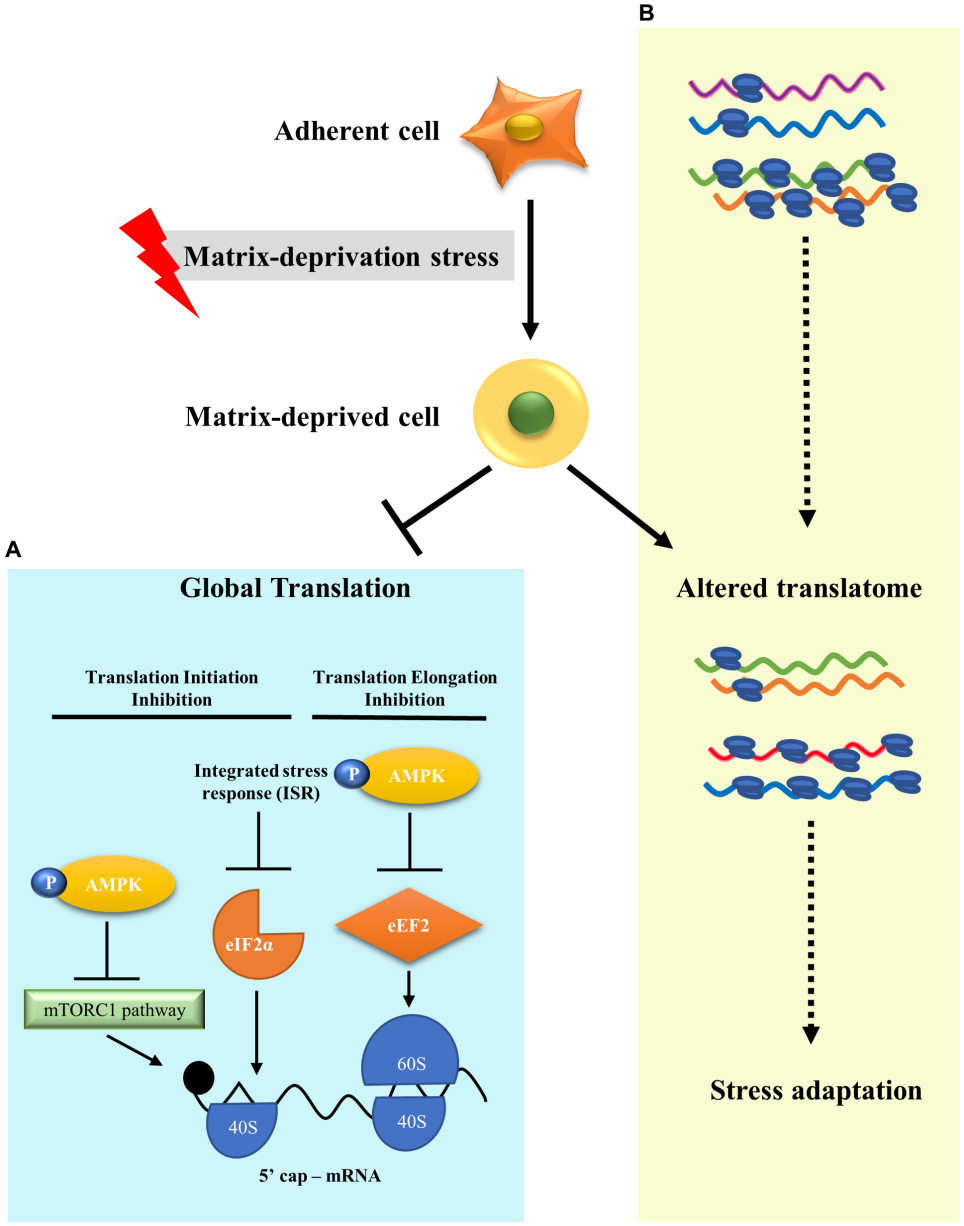
A model to show translational reprogramming in matrix-deprived breast cancer cell. **(A)** We show the suppression of global protein synthesis in matrix-detached cells by the inhibition of both translational initiation and elongation processes via activation of several stress-response pathways. **(B)** We also depict alteration of the translatome between adherent and matrix-deprived cells which may serve as a stress adaptation response.

Metastasis leads to 90% of cancer deaths (68). In order to metastasise, cancer cells must survive detachment and adapt to matrix-deprivation stress. Cells are known to modulate translation as an effective adaptation for energy conservation in response to stresses (32). However, translational reprogramming of cancer cells under the stress of matrix-deprivation is poorly explored. Here, we have shown that matrix-deprived breast cancer cells repress protein synthesis at initiation and elongation, significantly inhibiting global protein synthesis (Figure 4A). Yet, we observed minimal translation being sustained, suggesting the possibility of selective translation as an adaptive stress response (Figure 4B). Integrated omics analysis revealed the enrichment of putative candidate proteins with roles in cancer progression and/or stress response. These results broaden our understanding of matrix-deprivation as a stress encountered by cancer cells to facilitate metastasis.

Translation initiation is the rate-limiting step of protein synthesis and, thus, is targeted for regulation (26). It is regulated by both UPR and mTORC1 pathways in response to various stresses. Here, we report a novel observation of the phosphorylation of eIF2α under matrix-deprivation stress in breast cancer cells. Activation of the PERK-eIF2α axis increases stress tolerance, thus aiding survival in dormant disseminated tumour cells (69, 70), while its inhibition induces apoptosis in pancreatic cancer cells (71, 72), highlighting its importance in cellular homeostasis. We also demonstrate the inhibition of mTORC1 pathway during matrix-deprivation, thus, substantiating a previously reported observation (17). Its inhibition leads to global attenuation of cap-dependent translation (73, 74). Under several stresses, a pro-survival role for AMPK activation and subsequent mTORC1 inhibition is well known (47, 75). We have previously reported AMPK activation in breast cancer cells under matrix-deprivation (13, 20, 21), also corroborated in the present study. With our current data on mTORC1 inhibition, the AMPK-mTORC1 axis is implicated as a stress-response pathway in matrix-deprived breast cancer cells.

Phosphorylation of eEF2, a key translational regulator, by its upstream kinase eEF2K leads to its inactivation and stalling of translation elongation (76). Here, we demonstrate another novel observation of an increased phosphorylation of eEF2 under matrix-deprivation stress. AMPK is known to phosphorylate and activate eEF2K during multiple stresses (26, 77), and we have demonstrated AMPK activation in response to matrix detachment. Intriguingly, p70S6K directly phosphorylates and negatively regulates the activity of eEF2K (78). However, independent of both AMPK and mTORC1, eEF2K activation was also shown to lead to decreased protein synthesis and subsequent cancer cell death (79). Thus, tight control of translation elongation might be crucial in mediating adaptive stress-response.

Literature shows that global reduction of translation mediated by the activation of the ISR pathway is critical for the maintenance of tumour dormancy in the disseminated tumour cells (48). Additionally, deregulation of protein synthesis confers survival advantage in patient-derived breast CTCs, resulting in metastasis and poor clinical outcome (80). In our study, we present for the first time, the activation of aforementioned stress-response pathways in matrix-deprived patient-derived breast cancer cells. Discerning the stress responses under matrix-deprivation would help to further elucidate metastasis.

Activation of these canonical stress-response pathways culminates with the inhibition of translation. Our study offers one of the first comprehensive evaluations into translational repression under matrix-deprivation. Dramatic reduction in puromycylated nascent proteins in matrix-detached cells was also corroborated with the decline of polysome peaks and the concomitant increase in the monosome peaks. Such translational attenuation is also reported during other cellular stresses (51–53, 60). We also observed minimal translation being sustained under stress, which indicated the possibility of alternate translational mechanisms in matrix-deprived cells.

During energy stress, protein synthesis is streamlined towards the translation of selective mRNAs through alternate modes, which may be involved in stress survival (58, 81, 82). Selective mRNA translation is yet another uninvestigated concept in the field of matrix-deprivation stress. ATF4, translated under stress via the upstream ORF mechanism, is a major example of preferential mRNA translation (56). With respect to matrix-deprivation, an ATF4-mediated pro-survival role against anoikis is also reported in multiple cancer types (29, 83–85). We also demonstrate an upregulation of ATF4 in matrix-deprived breast cancer cells, thus indicating both the activation of ISR pathway as well as existence of altered translation. To obtain preferential mRNA translation dynamics under matrix-deprivation, our preliminary integrated omics analysis revealed putative candidate proteins that are altered at the protein level, whose mRNA levels remain unchanged. Based on this analysis, we report a novel observation of a significant increase in CNBP protein expression in matrix-deprived breast cancer cells. The same was also observed in patient tumour samples and patient-derived matrix-deprived cells. However, it is unknown whether the observed changes in protein levels are a reflection of their stability or translational reprogramming under matrix-deprivation stress. Further research into mRNAs that are translationally enriched in polysomes under matrix-deprivation and their integration with omics analysis would allow the identification of definite translational upregulation.

In summary, when breast cancer cells are subjected to matrix-deprivation, they display canonical hallmarks of stress response, notably inhibition of translation factors and mTORC1 pathway, resulting in global translational repression. We also suggest that detached breast cancer cells might potentially use an altered translatome to aid their survival. A deeper exploration of the residual translation occurring within matrix-deprived cancer cells would illuminate the mechanisms of stress adaptation during metastasis and identify novel targets to prevent cancer spread.

## Data availability statement

The RNA-Seq data has been deposited in NCBI Gene Expression Omnibus (GEO) under Accession code GSE234460. Proteomics data in this article, not yet fully analysed, is available with the authors and will be provided to any qualified researcher without undue reservation.

## Author contributions

SW conceived, designed and performed experiments, interpreted results, assembled data, undertook statistical analyses, and manuscript writing. SS helped with experiments, data collection, and manuscript writing. AC performed the RNA sequencing analysis and integrated omics analysis. AR was involved with the study design and conceptualisation, data interpretation, fund acquisition, and manuscript preparation. All authors read and approved the final version of the manuscript. All authors contributed to the article and approved the submitted version.

## Funding

This work was supported by the Wellcome Trust DBT India Alliance (500112-Z-09-Z to AR) and, funds from the DBT-IISc partnership programme of the Department of Biotechnology, Govt of India. Infrastructural support from the UGC to the Department of DBG is also acknowledged.

## Conflict of interest

The authors declare that the research was conducted in the absence of any commercial or financial relationships that could be construed as a potential conflict of interest.

## Publisher’s note

All claims expressed in this article are solely those of the authors and do not necessarily represent those of their affiliated organizations, or those of the publisher, the editors and the reviewers. Any product that may be evaluated in this article, or claim that may be made by its manufacturer, is not guaranteed or endorsed by the publisher.

## Footnotes

^1^https://software.broadinstitute.org/morpheus

## References

[1] Wilkinson L Gathani T. Understanding breast cancer as a global health concern. Br J Radiol. (2022) 95:7–9. doi: 10.1259/bjr.20211033 PMC8822551 34905391

[2] Ahmad A. Breast cancer metastasis and drug resistance: challanges and progress. Springer. (2019). 1–413

[3] Valastyan S Weinberg RA. Tumor metastasis: molecular insights and evolving paradigms. Cells. (2011) 147:275–92. doi: 10.1016/j.cell.2011.09.024, PMID: 22000009 PMC3261217

[4] Schoen FJ Mitchell RN. Tissues, the extracellular matrix, and cell–biomaterial interactions In: . Biomaterials science: an introduction to materials. 3rd ed: Academic Press (2013). 452–74.

[5] Frisch SM Francis H. Disruption of epithelial cell-matrix interactions induces apoptosis. J Cell Biol. (1994) 124:619–26. doi: 10.1083/jcb.124.4.619, PMID: 8106557 PMC2119917

[6] Guadamillas MC Cerezo A Del Pozo MA. Overcoming anoikis – pathways to anchorage-independent growth in cancer. Q J Microsc Sci. (2011) 124:3189–97. doi: 10.1242/jcs.072165 21940791

[7] Han H-J Sung JY Kim SH Yun UJ Kim H Jang EJ. Fibronectin regulates anoikis resistance via cell aggregate formation. Cancer Lett. (2021) 508:59–72. doi: 10.1016/j.canlet.2021.03.011, PMID: 33771684

[8] Yu Y Song Y Cheng L Chen L Liu B Lu D. CircCEMIP promotes anoikis-resistance by enhancing protective autophagy in prostate cancer cells. J Exp Clin Cancer Res. (2022) 41:188–17. doi: 10.1186/s13046-022-02381-7, PMID: 35655258 PMC9161511

[9] Hu P Li S Tian N Wu F Hu Y Li D. Acidosis enhances the self-renewal and mitochondrial respiration of stem cell-like glioma cells through CYP24A1-mediated reduction of vitamin D. Cell Death Dis. (2019) 10:25–14. doi: 10.1038/s41419-018-1242-1, PMID: 30631035 PMC6328565

[10] Wang C Shao L Pan C Ye J Ding Z Wu J. Elevated level of mitochondrial reactive oxygen species via fatty acid β-oxidation in cancer stem cells promotes cancer metastasis by inducing epithelial-mesenchymal transition. Stem Cell Res Ther. (2019) 10:1–16. doi: 10.1186/s13287-019-1265-2 31196164 PMC6567550

[11] Adeshakin FO Adeshakin AO Afolabi LO Yan D Zhang G Wan X. Mechanisms for modulating Anoikis resistance in cancer and the relevance of metabolic reprogramming. Front Oncol. (2021) 11:1–21. doi: 10.3389/fonc.2021.626577 PMC8039382 33854965

[12] Hardie DG Ross FA Hawley SA. AMPK: a nutrient and energy sensor that maintains energy homeostasis. Mol Cell Biol. (2012) 13:251–62. doi: 10.1038/nrm3311 PMC5726489 22436748

[13] Hindupur SK Balaji SA Saxena M Pandey S Sravan GS Heda N. Identification of a novel AMPK-PEA15 axis in the anoikis-resistant growth of mammary cells. Breast Cancer Res. (2014) 16:1–16. doi: 10.1186/s13058-014-0420-z PMC4303232 25096718

[14] Penugurti V Mishra YG Manavathi B. AMPK: an odyssey of a metabolic regulator, a tumor suppressor, and now a contextual oncogene. Biochim Biophys Acta. (2022) 1877:188785. doi: 10.1016/j.bbcan.2022.188785, PMID: 36031088

[15] Zhao M Finlay D Kwong E Liddington R Viollet B Sasaoka N. Cell adhesion suppresses autophagy via Src/FAK-mediated phosphorylation and inhibition of AMPK. Cell Signal. (2022) 89:110170. doi: 10.1016/j.cellsig.2021.110170, PMID: 34673141 PMC8602780

[16] Jeon S-M Chandel NS Hay N. AMPK regulates NADPH homeostasis to promote tumour cell survival during energy stress. Nat Cell Biol. (2012) 485:661–5. doi: 10.1038/nature11066 PMC3607316 22660331

[17] Ng TL Leprivier G Robertson MD Chow C Martin MJ Laderoute KR. The AMPK stress response pathway mediates anoikis resistance through inhibition of mTOR and suppression of protein synthesis. Cell Death Differ. (2012) 19:501–10. doi: 10.1038/cdd.2011.119, PMID: 21941369 PMC3278733

[18] Guo P Qiu Y Ma X Li T Ma X Zhu L. Tripartite motif 31 promotes resistance to anoikis of hepatocarcinoma cells through regulation of p53-AMPK axis. Exp Cell Res. (2018) 368:59–66. doi: 10.1016/j.yexcr.2018.04.013, PMID: 29665353

[19] Jin L Chun J Pan C Kumar A Zhang G Ha Y. The PLAG1-GDH1 Axis promotes Anoikis resistance and tumor metastasis through CamKK2-AMPK signaling in LKB1-deficient lung cancer. Mol Cell. (2018) 69:87–99.e7. doi: 10.1016/j.molcel.2017.11.025, PMID: 29249655 PMC5777230

[20] Sundararaman A Amirtham U Rangarajan A. Calcium-oxidant signaling network regulates AMP-activated protein kinase (AMPK) activation upon matrix deprivation. J Biol Chem. (2016) 291:14410–29. doi: 10.1074/jbc.M116.731257, PMID: 27226623 PMC4938166

[21] Saha M Kumar S Bukhari S Balaji SA Kumar P Hindupur SK. AMPK-AKT double negative feedback loop in breast cancer cells regulates their adaptation to matrix deprivation. Cancer Res. (2018) 78:1497. doi: 10.1158/0008-5472.CAN-17-2090 29339542 PMC6033311

[22] Xiao T Xu Z Zhou Y Zhang H Geng J Liang Y. Loss of TP53I11 enhances the extracellular matrix-independent survival by promoting activation of AMPK. IUBMB Life. (2019) 71:183–91. doi: 10.1002/iub.1949, PMID: 30376610

[23] Zhang P Song Y Sun Y Li X Chen L Yang L. AMPK/GSK3b/b-catenin cascade-triggered overexpression of CEMIP promotes migration and invasion in anoikis-resistant prostate cancer cells by enhancing metabolic reprogramming. FASEB J. (2018) 32:3924–35. doi: 10.1096/fj.201701078R, PMID: 29505302

[24] Buttgereit F Brand MD. A hierarchy of ATP-consuming processes in mammalian cells. Biochem J. (1995) 312:163–7. doi: 10.1042/bj3120163, PMID: 7492307 PMC1136240

[25] Xu Y Ruggero D. The role of translation control in tumorigenesis and its therapeutic implications. Annu Rev Cancer Biol. (2020) 4:437–57. doi: 10.1146/annurev-cancerbio-030419-033420

[26] Leprivier G Rotblat B Khan D Jan E Sorensen PH. Stress-mediated translational control in cancer cells. Biochim Biophys Acta. (2015) 1849:845–60. doi: 10.1016/j.bbagrm.2014.11.002, PMID: 25464034

[27] Avivar-Valderas A Salas E Bobrovnikova-Marjon E Diehl JA Nagi C Debnath J. PERK integrates autophagy and oxidative stress responses to promote survival during extracellular matrix detachment. Mol Cell Biol. (2011) 31:3616–29. doi: 10.1128/MCB.05164-11, PMID: 21709020 PMC3165554

[28] Avivar-Valderas A Bobrovnikova-Marjon E Alan Diehl J Bardeesy N Debnath J Aguirre-Ghiso JA. Regulation of autophagy during ECM detachment is linked to a selective inhibition of mTORC1 by PERK. Oncogene. (2013) 32:4932–40. doi: 10.1038/onc.2012.512 23160380 PMC3600386

[29] Dey S Sayers CM Verginadis II Lehman SL Cheng Y Cerniglia GJ. ATF4-dependent induction of heme oxygenase 1 prevents anoikis and promotes metastasis. J Clin Invest. (2015) 125:2592–608. doi: 10.1172/JCI78031, PMID: 26011642 PMC4563676

[30] Jinagal SL Shekhar P Chandra K Mutnuru SA Ramanan N Foretz M. Prolactin-induced AMPK stabilizes alveologenesis and lactogenesis through regulation of STAT5 signaling. *bioRxiv* [Internet] (2022), 2022.02.15.480514. Available at: http://biorxiv.org/content/early/2022/02/16/2022.02.15.480514.abstract

[31] Saxena M Balaji SA Deshpande N Ranganathan S Pillai DM Hindupur SK. AMP-activated protein kinase promotes epithelial-mesenchymal transition in cancer cells through Twist1 upregulation. J Cell Sci. (2018) 131:jcs208314. doi: 10.1242/jcs.208314, PMID: 29950484 PMC6080604

[32] Micalizzi DS Ebright RY Haber DA Maheswaran S. Translational regulation of cancer metastasis. Cancer Res. (2021) 81:517–24. doi: 10.1158/0008-5472.CAN-20-2720, PMID: 33479028 PMC7854484

[33] Hardie DG Schaffer BE Brunet A. AMPK: an energy-sensing pathway with multiple inputs and outputs. Trends Cell Biol. (2016) 26:190–201. doi: 10.1016/j.tcb.2015.10.013 26616193 PMC5881568

[34] Wek RC Jiang H-Y Anthony TG. Coping with stress: eIF2 kinases and translational control. Biochem Soc Trans. (2006) 34:7–11. doi: 10.1042/BST0340007, PMID: 16246168

[35] Gordiyenko Y Llácer JL Ramakrishnan V. Structural basis for the inhibition of translation through eIF2α phosphorylation. Nat Commun. (2019) 10:2640–11. doi: 10.1038/s41467-019-10606-1, PMID: 31201334 PMC6572841

[36] Leprivier G Remke M Rotblat B Dubuc A Mateo ARF Kool M. The eEF2 kinase confers resistance to nutrient deprivation by blocking translation elongation. Cell. (2013) 153:1064–79. doi: 10.1016/j.cell.2013.04.055 23706743 PMC4395874

[37] Aramburu J Ortells MC Tejedor S Buxadé M López-Rodríguez C. Transcriptional regulation of the stress response by mTOR. Sci STKE. (2014) 7:re2. doi: 10.1126/scisignal.2005326 24985347

[38] Kimball SR. Interaction between the AMP-activated protein kinase and mTOR signaling pathways. Med Sci Sports Exerc. (2006) 38:1958–64. doi: 10.1249/01.mss.0000233796.16411.13 17095930

[39] Chiang GG Abraham RT. Phosphorylation of mammalian target of rapamycin (mTOR) at Ser-2448 is mediated by p70S6 kinase. J Biol Chem. (2005) 280:25485–90. doi: 10.1074/jbc.M501707200, PMID: 15899889

[40] Inoki K Kim J Guan K-L. AMPK and mTOR in cellular energy homeostasis and drug targets. Annu Rev Pharmacol. (2012) 52:381–400. doi: 10.1146/annurev-pharmtox-010611-134537 22017684

[41] Gwinn DM Shackelford DB Egan DF Mihaylova MM Mery A Vasquez DS. AMPK phosphorylation of raptor mediates a metabolic checkpoint. Cells. (2008) 30:214–26. doi: 10.1016/j.molcel.2008.03.003 PMC2674027 18439900

[42] Hornberger TA Sukhija KB Wang XR Chien S. mTOR is the rapamycin-sensitive kinase that confers mechanically-induced phosphorylation of the hydrophobic motif site Thr(389) in p70S6k. FEBS Lett. (2007) 581:4562–6. doi: 10.1016/j.febslet.2007.08.045, PMID: 17825298 PMC2084087

[43] Thomas G. An encore for ribosome biogenesis in the control of cell proliferation. Nat Cell Biol. (2000) 2:E71–2. doi: 10.1038/35010581, PMID: 10806485

[44] Jastrzebski K Hannan KM Tchoubrieva EB Hannan RD Pearson RB. Coordinate regulation of ribosome biogenesis and function by the ribosomal protein S6 kinase, a key mediator of mTOR function. Growth Factors. (2007) 25:209–26. doi: 10.1080/08977190701779101 18092230

[45] Proud CG. Regulation of mammalian translation factors by nutrients. Eur J Biochem. (2002) 269:5338–49. doi: 10.1046/j.1432-1033.2002.03292.x 12423332

[46] Iadevaia V Huo Y Zhang Z Foster LJ Proud CG. Roles of the mammalian target of rapamycin, mTOR, in controlling ribosome biogenesis and protein synthesis. Biochem Soc Trans. (2012) 40:168–72. doi: 10.1042/BST20110682, PMID: 22260684

[47] Heberle AM Prentzell MT van Eunen K Bakker BM Grellscheid SN Thedieck K. Molecular mechanisms of mTOR regulation by stress. Mol Cell Oncol. (2015) 2:e970489. doi: 10.4161/23723548.2014.970489 27308421 PMC4904989

[48] Pakos-Zebrucka K Koryga I Mnich K Ljujic M Samali A Gorman AM. The integrated stress response. EMBO Rep. (2016) 17:1374–95. doi: 10.15252/embr.201642195, PMID: 27629041 PMC5048378

[49] Lindqvist LM Tandoc K Topisirovic I Furic L. Cross-talk between protein synthesis, energy metabolism and autophagy in cancer. Curr Opin Genet Dev. (2018) 48:104–11. doi: 10.1016/j.gde.2017.11.003, PMID: 29179096 PMC5869074

[50] Schmidt EK Clavarino G Ceppi M Pierre P. SUnSET, a nonradioactive method to monitor protein synthesis. Nat Methods. (2009) 6:275–7. doi: 10.1038/nmeth.1314, PMID: 19305406

[51] Shenton D Smirnova JB Selley JN Carroll K Hubbard SJ Pavitt GD. Global Translational Responses to Oxidative Stress Impact upon Multiple Levels of Protein Synthesis. J Biol Chem. (2006) 281:29011–21. doi: 10.1074/jbc.M601545200 16849329

[52] Thomas JD Johannes GJ. Identification of mRNAs that continue to associate with polysomes during hypoxia. RNA. (2007) 13:1116–31. doi: 10.1261/rna.534807, PMID: 17488873 PMC1894931

[53] Nagarajan S Grewal SS. An investigation of nutrient-dependent mRNA translation in Drosophila larvae. Biol Open. (2014) 3:1020–31. doi: 10.1242/bio.20149407 25305039 PMC4232759

[54] Chassé H Boulben S Costache V Cormier P Morales J. Analysis of translation using polysome profiling. Nucl Acids Res. (2017) 45:e15. doi: 10.1093/nar/gkw907, PMID: 28180329 PMC5388431

[55] Behrmann E Loerke J Budkevich TV Yamamoto K Schmidt A Penczek PA. Structural snapshots of actively translating human ribosomes. Cells. (2015) 161:845–57. doi: 10.1016/j.cell.2015.03.052, PMID: 25957688 PMC4432480

[56] Vattem KM Wek RC. Reinitiation involving upstream ORFs regulates ATF4 mRNA translation in mammalian cells. Proc Natl Acad Sci. (2004) 101:11269–74. doi: 10.1073/pnas.0400541101, PMID: 15277680 PMC509193

[57] Wortel IMN van der Meer LT Kilberg MS van Leeuwen FN. Surviving stress: modulation of ATF4-mediated stress responses in normal and malignant cells. Trends Endocrinol Metab. (2017) 28:794–806. doi: 10.1016/j.tem.2017.07.003, PMID: 28797581 PMC5951684

[58] Liu B Qian SB. Translational reprogramming in cellular stress response. Wiley Interdiscip Rev RNA. (2014) 5:301–5. doi: 10.1002/wrna.1212, PMID: 24375939 PMC3991730

[59] Balukoff NC Ho JJD Theodoridis PR Wang M Bokros M Llanio LM. A translational program that suppresses metabolism to shield the genome. Nat Commun. (2020) 11:1–15. doi: 10.1038/s41467-020-19602-2, PMID: 33188200 PMC7666154

[60] Krokowski D Jobava R Szkop KJ Chen CW Fu X Venus S. Stress-induced perturbations in intracellular amino acids reprogram mRNA translation in osmoadaptation independently of the ISR. Cell Rep. (2022) 40:111092. doi: 10.1016/j.celrep.2022.111092, PMID: 35858571 PMC9491157

[61] Yang F Hu A Li D Wang J Guo Y Liu Y. Circ-HuR suppresses HuR expression and gastric cancer progression by inhibiting CNBP transactivation. Mol Cancer. (2019) 18:1–16. doi: 10.1186/s12943-019-1094-z 31718709 PMC6852727

[62] D’Amico D Antonucci L di Magno L Coni S Sdruscia G Macone A. Non-canonical hedgehog/AMPK-mediated control of polyamine metabolism supports neuronal and medulloblastoma cell growth. Dev Cell. (2015) 35:21–35. doi: 10.1016/j.devcel.2015.09.008, PMID: 26460945 PMC4607931

[63] Liu H Fang Y Hou B Lin Q Zhang W Wang X. CircPACRGL promoted cell proliferation, migration and invasion as well as inhibited cell apoptosis in colorectal cancer via regulation of the miR-330-3p/CNBP axis. Mol Cell Biochem. (2022) 478:1633–44. doi: 10.1007/s11010-022-04543-9, PMID: 36459268

[64] Fan C Zhu X Zhou Q Wang W. CircFMN2 boosts sorafenib resistance in hepatocellular carcinoma cells via upregulating CNBP by restraining ubiquitination. J Oncol. (2022) 2022:1–9. doi: 10.1155/2022/2674163, PMID: 35909906 PMC9334069

[65] Jin GZ Zhang Y Cong WM Wu X Wang X Wu S. Phosphoglucomutase 1 inhibits hepatocellular carcinoma progression by regulating glucose trafficking. PLoS Biol. (2018) 16:1–27. doi: 10.1371/journal.pbio.2006483 PMC6193743 30335765

[66] Armas P Coux G Weiner AMJ Calcaterra NB. What’s new about CNBP? Divergent functions and activities for a conserved nucleic acid binding protein. Biochim Biophys Acta. (2021) 1865:129996. doi: 10.1016/j.bbagen.2021.129996, PMID: 34474118

[67] Rojas M Farr GW Fernandez CF Lauden L McCormack JC Wolin SL. Yeast Gis2 and its human ortholog CNBP are novel components of stress-induced RNP granules. PLoS One. (2012) 7:e52824. doi: 10.1371/journal.pone.0052824, PMID: 23285195 PMC3528734

[68] Chaffer CL Weinberg RA. A perspective on cancer cell metastasis. Science. (2011) 331:1559–64. doi: 10.1126/science.1203543 21436443

[69] Bartkowiak K Kwiatkowski M Buck F Gorges TM Nilse L Assmann V. Disseminated tumor cells persist in the bone marrow of breast cancer patients through sustained activation of the unfolded protein response. Cancer Res. (2015) 75:5367–77. doi: 10.1158/0008-5472.CAN-14-3728, PMID: 26573792

[70] Senft D Ronai ZA. Adaptive stress responses during tumor metastasis and dormancy. Trends Cancer. (2016) 2:429–42. doi: 10.1016/j.trecan.2016.06.004, PMID: 27868104 PMC5114008

[71] Palam LR Gore J Craven KE Wilson JL Korc M. Integrated stress response is critical for gemcitabine resistance in pancreatic ductal adenocarcinoma. Cell Death Dis. (2015) 6:1–13. doi: 10.1038/cddis.2015.264 PMC4632294 26469962

[72] El-Naggar AM Sorensen PH. Translational control of aberrant stress responses as a hallmark of cancer. J Pathol. (2018) 244:650–66. doi: 10.1002/path.5030, PMID: 29293271

[73] Reiling JH Sabatini DM. Stress and mTORture signaling. Oncogene. (2006) 25:6373–83. doi: 10.1038/sj.onc.1209889, PMID: 17041623

[74] Weber JD Gutmann DH. Deconvoluting mTOR biology. Cell Cycle. (2012) 11:236–48. doi: 10.4161/cc.11.2.19022, PMID: 22214661 PMC3293376

[75] Leprivier G Rotblat B. How does mTOR sense glucose starvation? AMPK is the usual suspect. Cell Death Discov. (2020) 6:27. doi: 10.1038/s41420-020-0260-9, PMID: 32351714 PMC7176732

[76] Zhu H Yang X Liu J Zhou L Zhang C Xu L. Eukaryotic elongation factor 2 kinase confers tolerance to stress conditions in cancer cells. Cell Stress Chaperones. (2015) 20:217–20. doi: 10.1007/s12192-014-0545-0, PMID: 25248493 PMC4326389

[77] Johanns M Pyrdit Ruys S Houddane A Vertommen D Herinckx G Hue L. Direct and indirect activation of eukaryotic elongation factor 2 kinase by AMP-activated protein kinase. Cell Signal. (2017) 36:212–21. doi: 10.1016/j.cellsig.2017.05.010, PMID: 28502587

[78] Wang X Li W Williams M Terada N Alessi DR Proud CG. Regulation of elongation factor 2 kinase by p90(RSK1) and p70 S6 kinase. EMBO J. (2001) 20:4370–9. doi: 10.1093/emboj/20.16.4370 11500364 PMC125559

[79] de Gassart A Demaria O Panes R Zaffalon L Ryazanov AG Gilliet M. PharmacologicaleEF2K activation promotes cell death and inhibits cancer progression. EMBO Rep. (2016) 17:1471–84. doi: 10.15252/embr.201642194, PMID: 27572820 PMC5048377

[80] Ebright RY Lee S Wittner BS Niederhoffer KL Nicholson BT Bardia A. Deregulation of ribosomal protein expression and translation promotes breast cancer metastasis. Science. (2020) 367:1468–73. doi: 10.1126/science.aay0939, PMID: 32029688 PMC7307008

[81] Lacerda R Menezes J Romão L. More than just scanning: the importance of cap-independent mRNA translation initiation for cellular stress response and cancer. Experientia. (2017) 74:1659–80. doi: 10.1007/s00018-016-2428-2 PMC11107732 27913822

[82] Sriram A Bohlen J Teleman AA. Translation acrobatics: how cancer cells exploit alternate modes of translational initiation. EMBO Rep. (2018) 19:e45947. doi: 10.15252/embr.201845947, PMID: 30224410 PMC6172470

[83] Mo H Guan J Mo L He J Wu Z Lin X. ATF4 regulated by MYC has an important function in anoikis resistance in human osteosarcoma cells. Mol Med Rep. (2018) 17:3658–66. doi: 10.3892/mmr.2017.8296, PMID: 29257326 PMC5802171

[84] Shi Z Yu X Yuan M Lv W Feng T Bai R. Activation of the PERK-ATF4 pathway promotes chemo-resistance in colon cancer cells. Sci Rep. (2019) 9:3210–8. doi: 10.1038/s41598-019-39547-x, PMID: 30824833 PMC6397152

[85] Yu Y Yang W Huang J Miao X Wang B Ren X. GPR120 induces regulatory dendritic cells by inhibiting HK2-dependent glycolysis to alleviate fulminant hepatic failure. Cell Death Dis. (2021) 13:1–13. doi: 10.1038/s41419-021-04394-0, PMID: 34911928 PMC8674251

