# Supplementary material for: Inhibition of protein translation under matrix-deprivation stress in breast cancer cells
Source: Front Med (Lausanne). 2023 Jun 22;10:1124514. doi: 10.3389/fmed.2023.1124514 (PMC10324034; doi:10.3389/fmed.2023.1124514)
Supplement: Supplementary file 1 [file Table_1.DOCX]

**Supplementary Material**

1. **Supplementary Table 1:** Details of top 5 upregulated proteins obtained in the differential translatome of MDA-MB-231 cells cultured in suspension with respect to attached condition for 24 hours

| **Protein Symbol** | **Full name** | **Biological function** |
| --- | --- | --- |
| **MRPL52** | Mitochondrial ribosomal protein L52 | Mitochondrial transport and Mitochondrial translation |
| **CNBP** | CCHC-type zinc finger nucleic acid binding protein | Transcriptional and translational regulation |
| **SLC7A11** | Solute carrier family 7 member 11 | Amino acid transport |
| **SUPT4H1** | SPT4 homolog, DSIF elongation factor subunit | Transcriptional regulation |
| **GATA6** | GATA binding protein 6 | Transcriptional regulation |

1. **Supplementary Table 2:** Details of top 5 downregulated proteins obtained in the differential translatome of MDA-MB-231 cells cultured in suspension with respect to attached condition for 24 hours.

| **Protein Symbol** | **Full name** | **Biological function** |
| --- | --- | --- |
| **FUT10** | Fucosyl transferase 10 | Metabolism |
| **C20orf16** | Spermine oxidase | Polyamine metabolism |
| **ZNF614** | Zinc finger protein 614 | Transcriptional regulation |
| **CFAP161** | Cilia and flagella assocaited protein 161 | Cilia motility |
| **FGD6** | FYVE, RhoGEF and PH domain containing 6 | Regulation of cytoskeleton |
