# Supplementary figures and images for: Inhibition of protein translation under matrix-deprivation stress in breast cancer cells
Source: Front Med (Lausanne). 2023 Jun 22;10:1124514. doi: 10.3389/fmed.2023.1124514 (PMC10324034; doi:10.3389/fmed.2023.1124514)

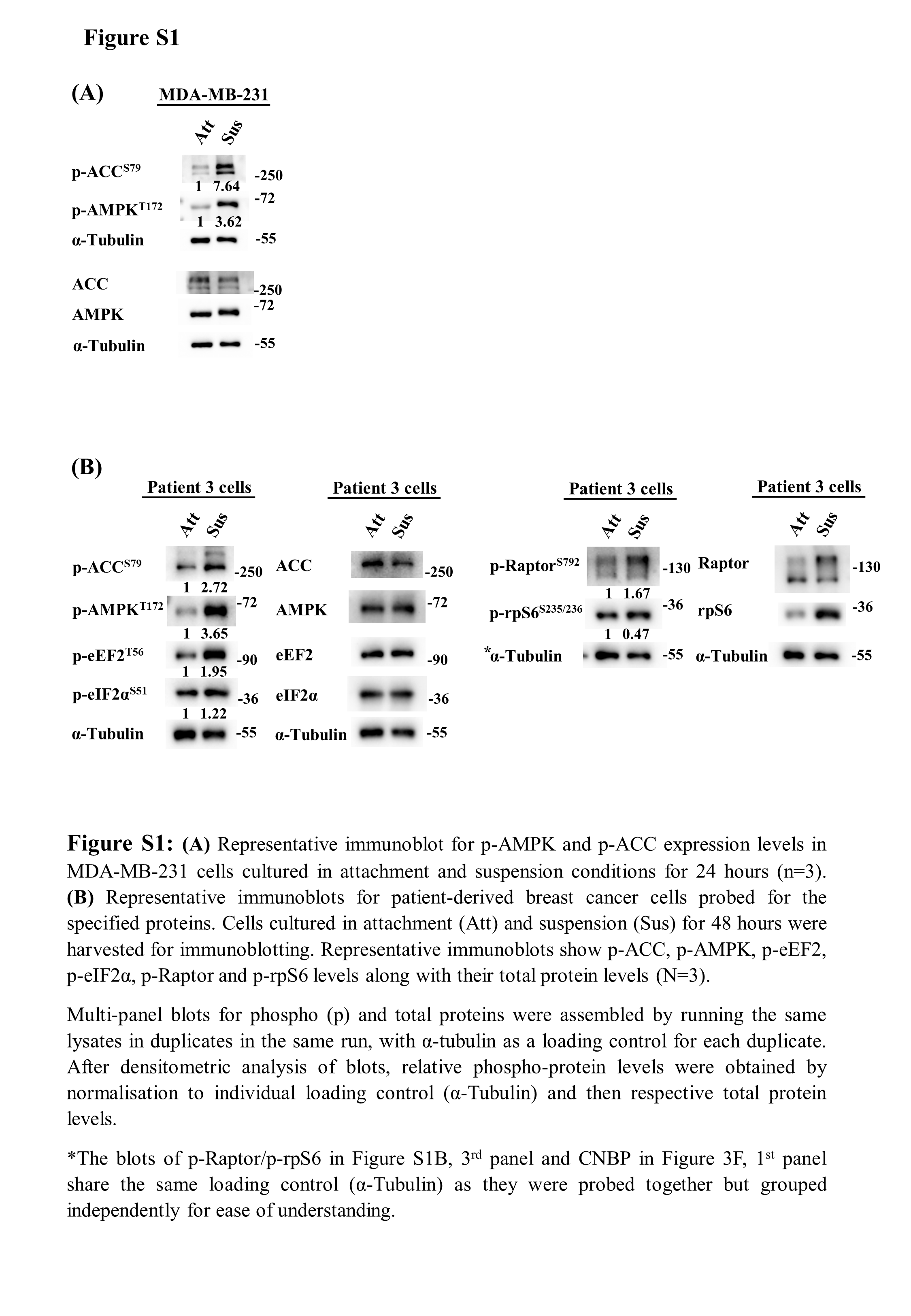

Supplement: Supplementary file 2 [file Image_1.tif]

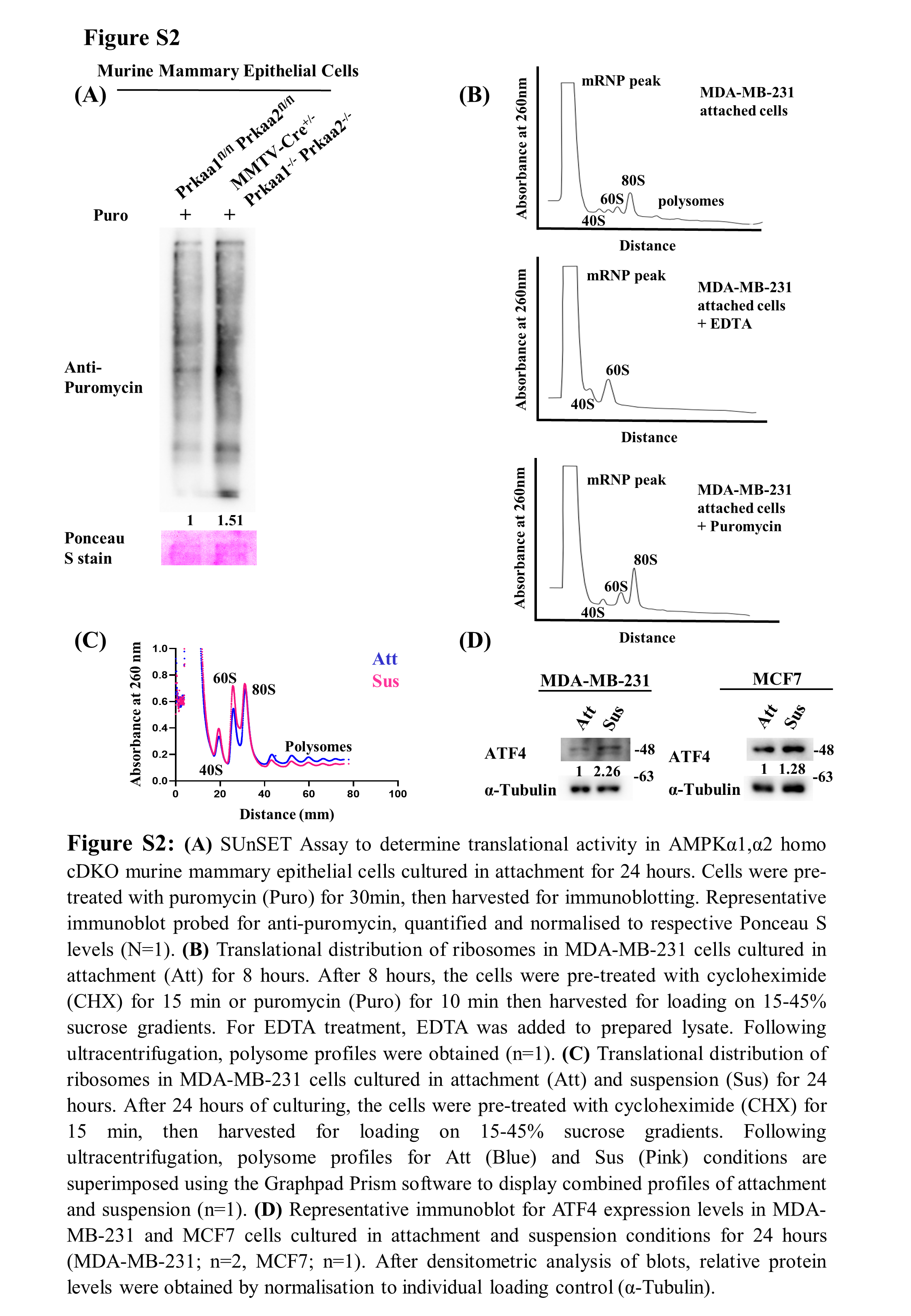

Supplement: Supplementary file 3 [file Image_2.TIF]
